# The Fyn–ADAP Axis: Cytotoxicity Versus Cytokine Production in Killer Cells

**DOI:** 10.3389/fimmu.2015.00472

**Published:** 2015-09-16

**Authors:** Zachary J. Gerbec, Monica S. Thakar, Subramaniam Malarkannan

**Affiliations:** ^1^Laboratory of Molecular Immunology and Immunotherapy, Blood Research Institute, Medical College of Wisconsin, Milwaukee, WI, USA; ^2^Department of Microbiology, Immunology and Molecular Genetics, Medical College of Wisconsin, Milwaukee, WI, USA; ^3^Department of Pediatrics, Medical College of Wisconsin, Milwaukee, WI, USA; ^4^Department of Medicine, Medical College of Wisconsin, Milwaukee, WI, USA

**Keywords:** Fyn, ADAP, SFK, signaling, structure–activity relationship

## Abstract

Lymphocyte signaling cascades responsible for anti-tumor cytotoxicity and inflammatory cytokine production must be tightly regulated in order to control an immune response. Disruption of these cascades can cause immune suppression as seen in a tumor microenvironment, and loss of signaling integrity can lead to autoimmunity and other forms of host-tissue damage. Therefore, understanding the distinct signaling events that exclusively control specific effector functions of “killer” lymphocytes (T and NK cells) is critical for understanding disease progression and formulating successful immunotherapy. Elucidation of divergent signaling pathways involved in receptor-mediated activation has provided insights into the independent regulation of cytotoxicity and cytokine production in lymphocytes. Specifically, the Fyn signaling axis represents a branch point for killer cell effector functions and provides a model for how cytotoxicity and cytokine production are differentially regulated. While the Fyn–PI(3)K pathway controls multiple functions, including cytotoxicity, cell development, and cytokine production, the Fyn–ADAP pathway preferentially regulates cytokine production in NK and T cells. In this review, we discuss how the structure of Fyn controls its function in lymphocytes and the role this plays in mediating two facets of lymphocyte effector function, cytotoxicity and production of inflammatory cytokines. This offers a model for using mechanistic and structural approaches to understand clinically relevant lymphocyte signaling.

## Introduction – Fyn, PI(3)K, and ADAP

Effector functions of lymphocytes are mediated through signaling cascades that transmit extracellular signals to intracellular response elements. These signaling pathways allow lymphocytes to respond to transformed or virally infected cells via cell-mediated cytotoxicity and inflammatory cytokine production. The identity of receptor–ligand interactions and downstream intracellular proteins responsible for inducing effector functions has been well established in NK and T cells. However, the divergent roles of signaling molecules in mediating distinct effector functions remain largely uncharacterized. Fyn is a membrane proximal, non-receptor tyrosine kinase responsible for initiating signaling cascades downstream of the TCR and multiple NK cell-activating receptors (1). By recruiting multiple downstream effector molecules, Fyn functions as a branch point for signaling divergence.

Fyn is one of nine members (Src, Fyn, Lyn, Lck, Fgr, Yes, Hck, Blk, and Frk) of the Src-family of non-receptor tyrosine kinases (SFKs) (2). Since the discovery of v-Src and its corresponding cellular protein c-Src, extensive study has characterized the pleiotropic nature of this tyrosine kinase family (3). SFKs participate in a myriad of cellular functions, including growth, differentiation, migration, adhesion, and in lymphocytes, cytotoxicity, and cytokine production (4). In T and NK cells, activation of Fyn induces binding to distinct signaling partners, such as PI(3)K-p85α (5, 6) and the lymphocyte-specific scaffold protein adhesion and degranulation-promoting adaptor protein (ADAP) (7). Recruitment of these substrates through Src-homology (SH) domains allows Fyn to initiate and regulate diverse lymphocyte effector functions.

Through its ability to recruit and phosphorylate the p85α subunit of PI(3)K, Fyn is able to activate signaling cascades responsible for a variety of cellular functions, including cell development, cell-mediated cytotoxicity, and production of inflammatory cytokines (8). Independent of the PI(3)K pathway, Fyn recruits and activates the ADAP signaling axis predominantly responsible for cytokine production in lymphocytes. Fyn–ADAP binding occurs exclusively in lymphocytes, and activation of this pathway is mediated by an interaction between the Fyn SH2 domain and the pYDGI motif of ADAP corresponding to residues 625–628 (9). This interaction induces translocation of ADAP to the membrane and stabilizes the protein. From the membrane, ADAP is able to mediate formation of the Carma1-Bcl10-Malt1 (CBM) complex critical for NF-κB and AP-1 activation (10–12). These transcription factors are critical for inflammatory cytokine and chemokine production downstream of the Fyn–ADAP interaction in effector cells (10–12).

Fyn therefore represents a node of signaling divergence for two primary outcomes of lymphocyte activation, cytotoxicity and inflammatory cytokine production. In this review, we discuss how the structure of Fyn allows it to act as a critical regulator of divergent signaling pathways and multiple effector functions in lymphocytes. We discuss conformational changes necessary for substrate recognition, specifically PI(3)K and ADAP, as well as control of enzymatic activity. We also discuss how protein structure allows Fyn to act as the exclusive mediator of the ADAP signaling cascade and resulting inflammatory cytokine production, thereby regulating a portion of the systemic immune response.

## Domain Structures of Fyn

Src-family of non-receptor tyrosine kinases are pleiotropic in nature and multiple family members, including Fyn, are ubiquitously expressed in multiple cell types. This allows them to control a variety of cellular functions and highlights the need for maintaining signaling integrity both in terms of enzymatic activity and substrate specificity (13). Across multiple cell types, this regulation is provided by the structure of SFKs which creates specificity that controls the substrates of the kinase domain and regulates enzymatic activity. In lymphocytes specifically, this allows Fyn to act as a branch point in regulating cytotoxicity and production of inflammatory cytokines (10–12, 14, 15). The structure of Fyn provides a potential mechanistic explanation for its ability to regulate these diverse effector functions in the context of effector-cell signaling. This is critical in activated lymphocytes because it enables differential regulation of effector functions downstream of a single protein.

**Figure 1:**
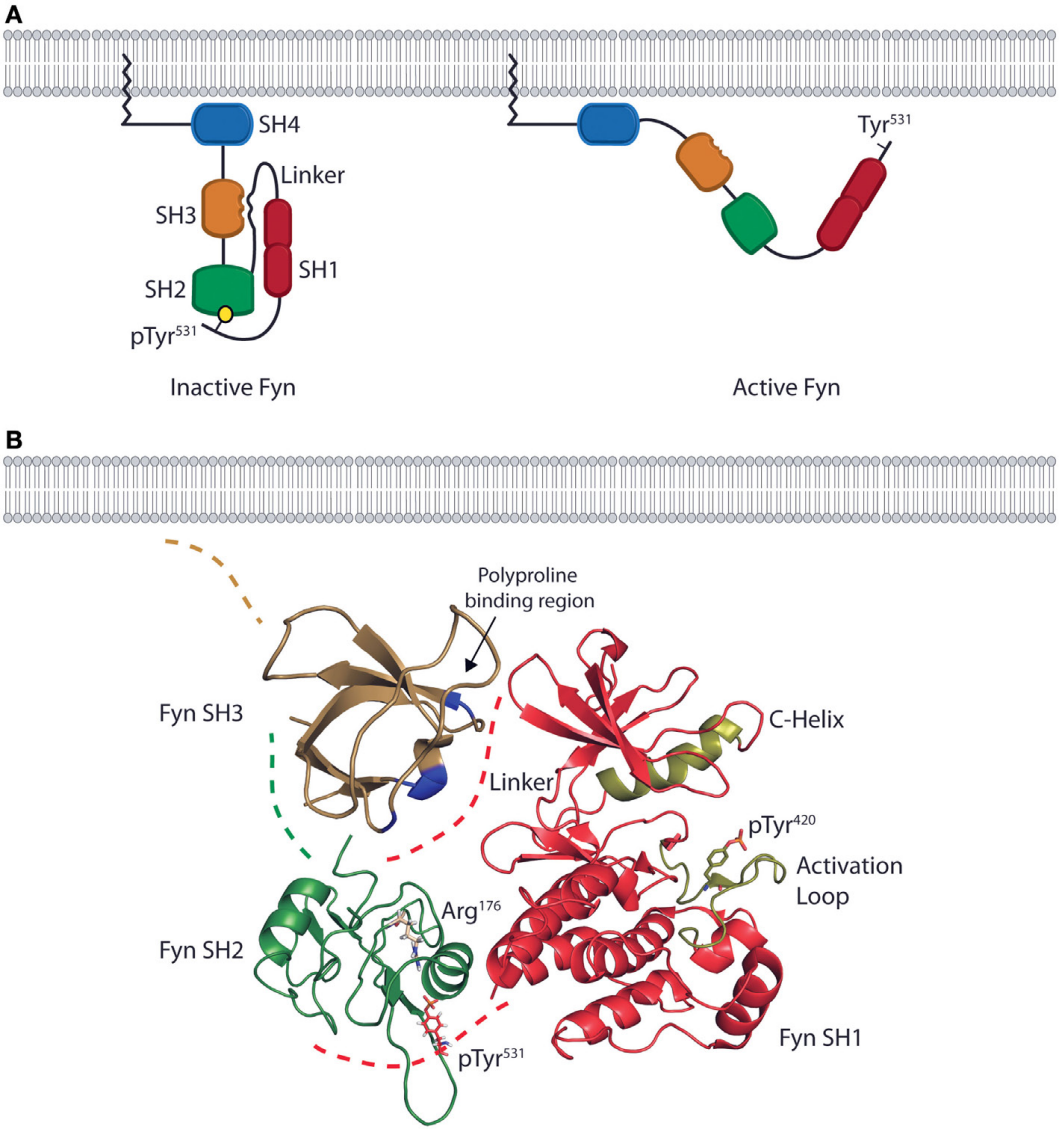
**The domain structure and intramolecular interactions of the Fyn kinase**. **(A)** Models of the two primary conformations of the Fyn kinase. The domain structure of Fyn consists of four SH domains. The relative positions of the domains are shown in both the inactive (left) and active (right) states. The N-terminal SH4/unique domain (blue) lies proximal to the membrane-anchoring myristoylation site. The SH4 domain is followed by the SH3 (gold) and SH2 (green) domains responsible for mediating interactions between Fyn and its target substrates. These domains are followed by a linker region that connects the SH2 domain to the bilobal SH1/kinase domain (red) responsible for enzymatic activity. The inactive form of the kinase is kept in a closed conformation by intramolecular interactions between the SH2 domain and a C-terminal phosphotyrosine as well as by an interaction between the SH3 domain and a polyproline helix in the linker region (left). The active conformation is adopted upon disruption of these interactions and opening of the kinase. In the active conformation, the SH2 and SH3 domains mediate protein–protein interactions, while the kinase domain phosphorylates downstream effectors (right). **(B)** Ribbon diagrams of the SH3, SH2, and SH1 domains show their relative positions in the inactive conformation. Positions are based on the structure of auto-inhibited Src (PDB deposition 2SRC), which has an identical domain arrangement. The blue high-lighted area of the SH3domain is the site of interaction with the linker region. The SH2 domain of Fyn (green) possesses an Arg residue in the second β-sheet (ArgβB5, Arg^176^) that is conserved across SFK family members. This Arg residue is the primary site of interaction with the phosphotyrosine on target substrates. In the inactive conformation, Arg^176^ is bound to the C-terminal inhibitory pTyr^531^ of Fyn. These intramolecular interactions place the SH2 and SH3 domains in a position to occlude the kinase domain (red) from substrate binding, thus preventing phosphate transfer. The ribbon diagram of the kinase domain is derived from PDB deposition 2DQ7, and the positions of the C-Helix and activation loop (gold) show the structure of the Fyn kinase domain complexed with staurosporine. To date, this is the only conformation of the Fyn kinase domain that has been characterized, and it is used here to demonstrate the intramolecular interactions that keep Fyn in an inactive state. The SH3 domain ribbon diagram is from PDB deposition 1NYG, and the SH2 ribbon diagram is from the PDB deposition 1AOT, and is representative of the SH2 domain bound to a phosphotyrosine-containing peptide.

The structure of Fyn is conserved across all cell types, and *in vitro* characterization of the protein structure combined with *in vivo* characterization of Fyn function in different cell types provides a basis for understanding structure-based Fyn regulation in lymphocytes. The quaternary structure of Fyn and the consecutive arrangement of the SH4, SH3, and SH2 domains control the function of the kinase domain (SH1) through substrate recognition and regulation of enzymatic activity (Figure 1A). The N-terminal SH4 domain of Fyn is co-translationally myristoylated on the N-terminal Gly^2^ residue and serves as a membrane targeting and anchoring sequence (Figure 1A). The SH4 domain also contains a non-conserved region that contributes to the specific functions of SFK family members. In the case of Fyn, this unique region contains two Cys residues (Cys^3^ and Cys^6^) that can be reversibly palmitoylated. Studies using fibroblast cell lines show that palmitoylation of these residues induces localization to lipid rafts in the plasma membrane and contributes to spatial control of signaling (16, 17). The SH4 domain of Fyn is followed by consecutive SH3 and SH2 domains responsible for mediating both intermolecular and intramolecular protein interactions (Figure 1A) (18). The SH3 domain consists of a β-barrel formed by five anti-parallel β-strands (19) and two loops that form a binding pocket for linear, Pro-rich peptides. The polyproline sequences of interacting partners form helical conformations that bind with aromatic side chains of the Fyn SH3 domain. The Fyn SH2 domain is characterized by a primary binding pocket and a specificity-determining region. These two sites interact with a phosphorylated Tyr and proximal residues on binding partners (20). The SH2 and SH3 domains allow Fyn to position substrates adjacent to the bilobal tyrosine kinase (SH1) domain at its C-terminus (Figure 1A) (4). Both the N- and C-terminal lobes of the SH1 domain contain regulatory sequences (the C-Helix in the N-terminal lobe and the activation loop in the C-terminal lobe) that control ATP-binding and phosphate transfer (21–23). The kinase domain also contains a regulatory Tyr (Tyr^420^) that is auto-phosphorylated upon activation of enzymatic activity. Collectively, the SH domains and quaternary structure of Fyn enable it to act in multiple facets of killer cell effector function by providing the substrate specificity and control of enzymatic activity required for regulation of divergent signaling.

## Intrinsic Regulation of Fyn Activation

*In vitro* characterization of SFK structures show that the function of Fyn is regulated by intramolecular interactions between the SH domains that prevent unnecessary activation under resting state. Structures of the full-length Fyn protein in both the inactive and active conformations remain to be elucidated; however, sequence homology and structural data of individual domains of Fyn and other SFKs provide a model for the intramolecular interactions that regulate Fyn activity in lymphocytes (Figure 1B). X-ray crystallographic structures of auto-inhibited Src and Hck reveal that prior to SFK activation, the kinase domain is kept in a closed conformation as a result of intramolecular binding between a phosphorylated Tyr (Tyr^531^ in Fyn) in the C-terminal regulatory region and the SH2 domain (21–23). Despite the presence of high-affinity peptide motifs on binding partners of Fyn, this interaction is likely maintained due to the quaternary structure of Fyn creating a high local concentration of the C-terminal inhibitory peptide (24–26). The SH3 domain of SFKs further stabilizes this conformation by binding to a polyproline helix in the linker region between the SH2 and kinase domains (Figure 1B) (21–23).

These intramolecular interactions force SFKs into an inactive conformation that results from the quaternary structure of Fyn. In this conformation, the SH3 domains lie proximal to the N-terminal lobe of the kinase domain, and the SH2 domains lie proximal to the C-terminal lobe as a result of interacting with the C-terminal phosphotyrosine residue (Figure 1B) (21–23, 27). This not only results in partial occlusion of the kinase domain due to the close proximity of the SH2 and SH3 domains, but it also positions the C-Helix and activation loop to prevent ATP and substrate binding (21–23). To corroborate these structural data, studies using multiple SFKs, including Fyn, show that loss of a functional SH2 domain or C-terminal regulatory region leads to aberrant kinase activity and cellular transformation (28–30). Together, both functional and structural data suggest maintaining the correct quaternary structure of Fyn, and the resultant intramolecular contacts, is required for the temporal regulation of signaling in lymphocytes.

## Extrinsic Regulation of Fyn Activation by Csk and PAG

**Figure 2:**
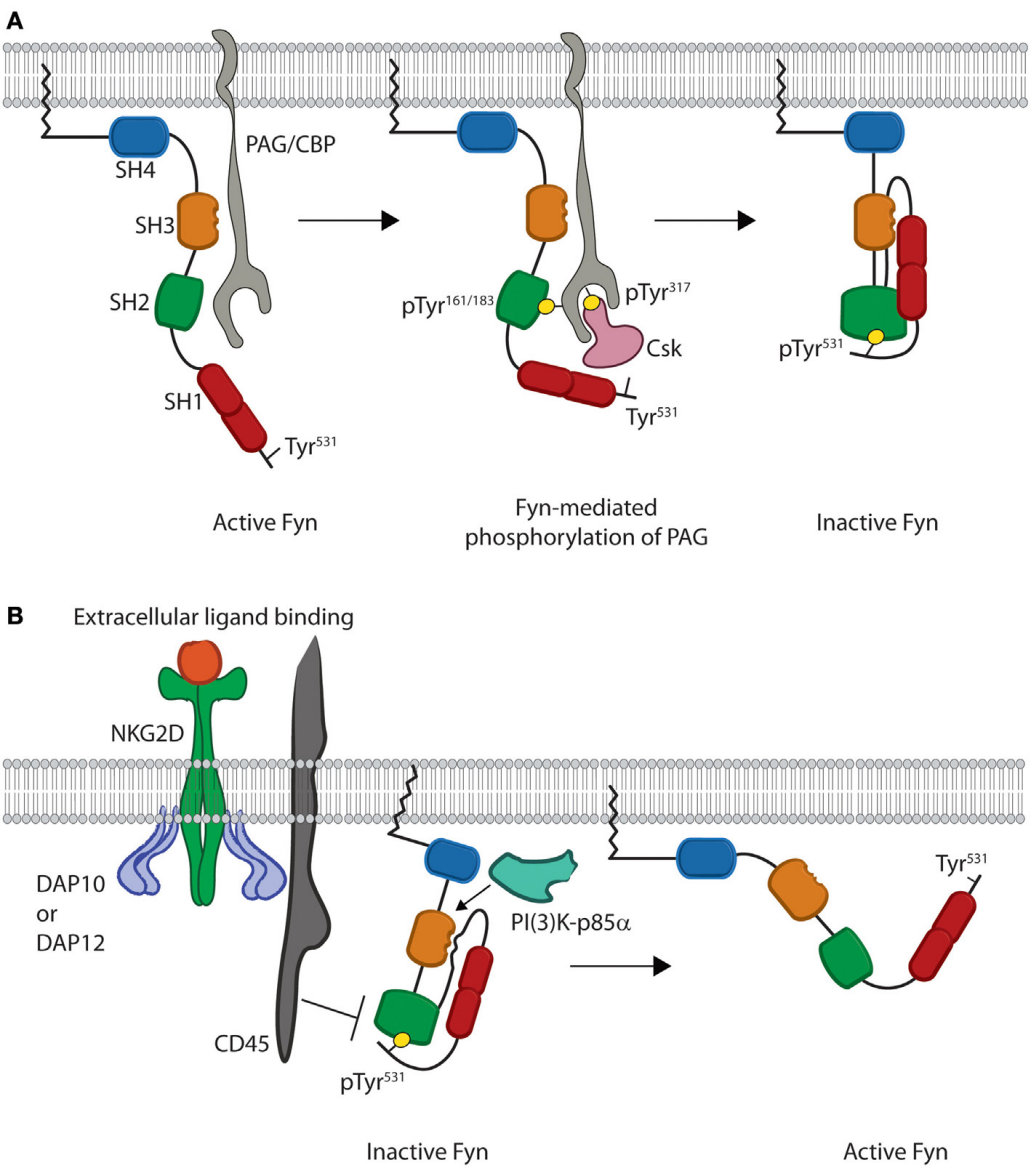
**Models for inhibition and activation of Fyn**. **(A)** Fyn regulation is reliant on phosphorylation of an inhibitory tyrosine, Tyr^531^, in the C-terminal regulatory region. This is initiated by binding of the SH3 domain of open Fyn to PAG/CBP (gray) (left). This leads to subsequent phosphorylation of tyrosine residues on PAG/CBP, and these tyrosine residues bind the SH2 domains of both Fyn and Csk (pink) (center). Csk then phosphorylates the C-terminal inhibitory Tyr^531^, which leads to intramolecular binding of the Fyn SH2 domain and disrupts the interaction of Fyn with PAG/CBP. Fyn is then released from PAG/CBP and remains anchored in the membrane as an inactive kinase molecule (right). **(B)** Fyn is locked in the inactive conformation until cellular activation leads to disruption of intramolecular binding. Stimulation through NKG2D leads to activation of the CD45 phosphatase (left). CD45 then removes the phosphate from the C-terminal inhibitory tyrosine and disrupts the intramolecular interactions responsible for keeping Fyn in a closed conformation. These interactions are also disrupted upon binding of substrates, such as PI(3)K-p85α, to the Fyn SH3 domain. This prevents the SH3 domain from binding to the linker region. Loss of these intramolecular interactions causes Fyn to adopt a more open conformation where the SH2 and SH3 domains are able to mediate substrate recruitment, while the kinase domain mediates enzymatic activity (right).

The quaternary structure of Fyn also provides extrinsic regulation that helps maintain Fyn in an inactive state as evidenced by studies in multiple cell types. When Fyn is co-translationally myristoylated, the carbon chain of the myristoyl group causes translocation of the protein to the plasma membrane (17). Once at the membrane, interaction of Fyn with an adaptor protein and regulatory kinase forces Fyn into a closed conformation (Figure 2A). These events are driven by processive phosphorylation, wherein all available sites of a given substrate are phosphorylated before the kinase dissociates.

Studies using T cells and mast cells show that at the membrane, Fyn first associates with the transmembrane adaptor phosphoprotein-associated with glycosphingolipid-enriched domains/c-Src kinase (Csk)-binding protein (PAG/CBP) (31–33). This interaction is mediated by binding of the SH3 domain of Fyn to a Pro-rich region of PAG between residues 131–137 as evidenced by *in vitro* studies using peptides derived from PAG (Figure 2A) (34). Along with controlling the protein–protein interaction, Solheim et al. used *in vitro* phosphorylation assays to show that this binding event actually increases the enzymatic activity of Fyn likely through a switch to a more active conformation (34).

The increased activity of Fyn next results in phosphorylation of either Tyr residue Tyr^163^ or Tyr^181^ of PAG, which initiates binding of the SH2 domain of Fyn (31–33). The resultant SH2–phosphotyrosine interaction then leads to processive phosphorylation of multiple Tyr residues, including Tyr^317^of PAG necessary for recruitment of the regulatory protein c-Src kinase (Csk) (Figure 2A) (33). Association of this kinase with PAG allows Csk to phosphorylate the C-terminal inhibitory tyrosine of Fyn. Finally, dissociation of Fyn from PAG then induces the inactive conformation of Fyn (Figure 2A) (33). This pathway demonstrates how protein structure allows for crosstalk between different domains and extrinsic control of kinase activity. Taken together, these data demonstrate how the quaternary structure creates an effective off switch for Fyn activity through extrinsic regulation. In lymphocytes, this allows Fyn to maintain an inactive conformation prior to receptor-mediated activation.

## Fyn Structure in Activation

The precise mechanism of Fyn activation in lymphocytes is yet to be fully characterized; however, several studies suggest that disruption of intramolecular interactions triggers Fyn to adopt an open conformation. Specifically, Fyn activity has been shown to increase following dephosphorylation of C-terminal inhibitory Tyr^531^ as well as substrate recruitment to the SH3 domain (Figure 2B). To date, the primary phosphatase implicated in Fyn activation is the transmembrane protein CD45. Murine NK cells from CD45-deficient mice show that cytokine production is reduced downstream of Ly49D, CD16, and NKG2D receptors following activation (35). Several studies using T and NK cells also suggest that CD45-mediated dephosphorylation leads to Fyn activation. Both murine and human T and NK cells deficient in CD45 show hyper-phosphorylation of Tyr^531^ of Fyn and impaired effector functions following receptor-mediated activation (35–39). Additionally, hyper-phosphorylation of Fyn prevents activation of PLC-γ1 and calcium flux downstream of CD3- and CD28-mediated stimulation (40, 41). These data suggest that CD45-mediated dephosphorylation is obligatory for Fyn activation in lymphocytes (42). *In vitro* enzyme kinetics assays also show that Fyn activation can be achieved through interaction of substrates with the SH3 domain (43, 44). Fyn-substrate binding results in displacement of the SH3 domain from the linker region thereby disrupting intramolecular interactions that keep Fyn in an inactive state (44–47). While the complete open structure of Fyn has not been elucidated, crystal structures of other SFKs suggest that disruption of these intramolecular interactions displaces the SH2 and SH3 domains from the kinase domain (Figure 2B) (48). These conformational changes eliminate steric hindrance and induce the active state of the Fyn kinase domain.

Crystal structures of Src and Lck show that movement of the SH3 and SH2 domains away from the kinase domain alters the helical conformation of the activation loop in the C-terminal lobe (Figure 1B) of the kinase domain (45–47). This allows for auto-phosphorylation of an activating Tyr^420^ in the kinase domain of Fyn. This creates an electrostatic interaction that moves the C-Helix of the N-terminal lobe (Figure 1B) into an active position where it is able to help place ATP for phosphate transfer (45–47). Despite the fact that the specific mechanism of Fyn activation in lymphocytes has not been characterized, these structural and functional data allow us to propose a model whereby Fyn undergoes a conformational switch due to CD45-mediated dephosphorylation and binding of PI(3)K-p85α to the SH3 domain. This results in disruption of intramolecular contacts, converting the kinase domain to an active conformation while opening the SH2 and SH3 domains to mediate substrate recruitment. These domains give Fyn the ability to recruit and phosphorylate distinct downstream proteins thereby initiating divergent signaling pathways.

## Fyn Recruits Distinct Signaling Partners to Regulate Multiple Cellular Functions

**Figure 3:**
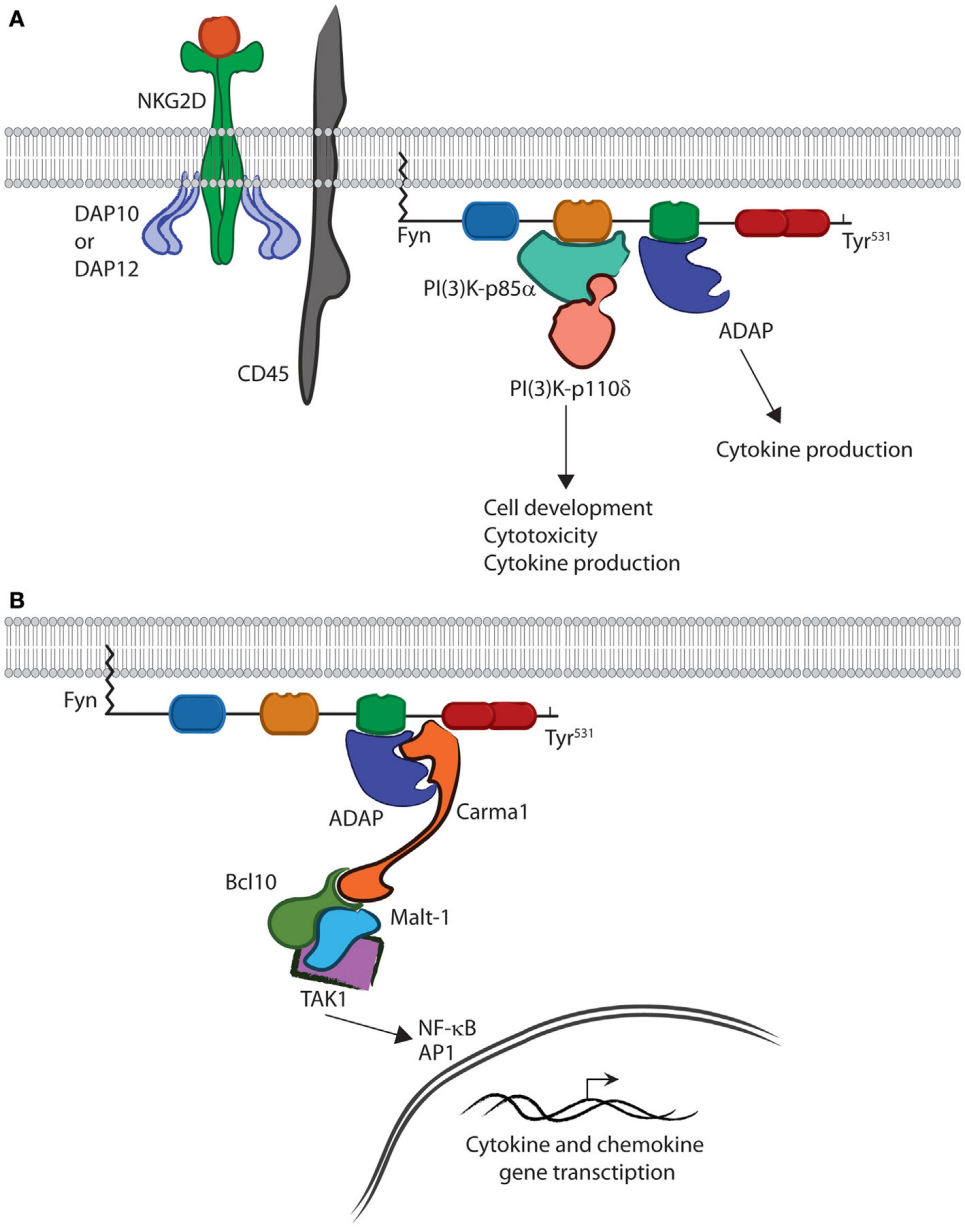
**Fyn is capable of activating divergent signaling cascades**. **(A)** In the active conformation, the SH2 and SH3 domains bind downstream effectors and activate divergent signaling cascades in lymphocytes. The interaction between PI(3)K-p85α and the SH3 domain of Fyn controls multiple aspects of lymphocyte effector functions. The SH2 domain of Fyn binds ADAP via the YDGI motif following tyrosine phosphorylation by Fyn. This stabilizes ADAP and initiates a unique set of lymphocyte functions, such as cytokine production. **(B)** ADAP translocation and stabilization leads to CBM complex formation. Carma1 binds to ADAP and initiates the recruitment of Bcl10 and Malt1. This leads to TAK1 binding and activation of its kinase activity. TAK1 then activates NF-κB or AP-1. Once activated, NF-κB and AP1 translocate to the nucleus where they induce transcription of cytokine and chemokine genes.

The SH3 domain of Fyn is able to bind the PI(3)K-p85α subunit and, through phosphorylation, initiate PI(3)K activity (Figure 3). Structural data show that a Pro-rich region corresponding to residues 91–104 of PI(3)K-p85α is able to adopt a helical conformation and bind the SH3 domain of Fyn (19). This binding leads to phosphorylation and activation of PI(3)K-p85α (49). These interactions occur downstream of receptor-mediated activation and are critical for lymphocyte effector function (50–52). Studies with PI(3)K-p85α-deficient murine NK cells show loss of cytokine production and anti-tumor cytotoxicity (53). In addition to these functional defects, loss of PI(3)K-p85α also impairs lineage commitment and terminal maturation (53). These data demonstrate the ability of Fyn to activate multiple lymphocyte effector functions through activation of PI(3)K signaling.

The quaternary structure of Fyn also allows it to activate a divergent signaling cascade primarily responsible for cytokine production in lymphocytes. Recent work has elucidated the role of the Fyn–ADAP axis in mediating inflammatory cytokine production in activated NK and T cells (10–12, 14). A unique pYDGI motif corresponding to residues 625–628 of ADAP facilitates its binding to the SH2 domain of Fyn (Figure 3). This stabilizes ADAP and allows it to bind the C-terminal region of Carma1, leading to CBM signalosome formation, NF-κB translocation, and cytokine production (14). Studies using NKG2D- and CD137-mediated activation of NK cells show that these pathways are turned on simultaneously as evidenced by PI(3)K-p85α phosphorylation and nuclear translocation of p65 (12). However, it remains unclear as to whether a single molecule of Fyn can bind both PI(3)K-p85α and ADAP concurrently (Figure 3). The fact that these pathways are mediated by different SH domains suggests that this type of regulation is possible. Structural analysis using multiple Fyn-binding targets may elucidate whether these molecules bind simultaneously or in a sequential manner.

The role of the Fyn SH2 domain in initiating the ADAP-CBM-Tak1 signaling cascade leading to cytokine production highlights the importance of the unique structure of the Fyn. Lack of ADAP in NK cells impairs only cytokine production but, not anti-tumor cytotoxicity (12). However, lack of Fyn alters both cytokine production and cytotoxicity, implying that Fyn initiates and regulates multiple signaling cascades that, despite being regulated by a single protein, are able to function independently. The structure of Fyn suggests that this is due to substrate control provided by the SH2 and SH3 domain, and in the case of the Fyn–ADAP axis, the SH2 domain specifically. To date, over 100 SH2 domain-containing proteins have been identified (54, 55). Despite this conservation, Fyn remains the sole kinase shown to be responsible for activation of ADAP-dependent cytokine production. This is due to structural differences creating unique binding specificities among protein family members and across all SH2 domain-containing proteins. Thus, the SH2 domain of Fyn provides an excellent example of how subtle structural differences create signaling specificity critical for regulation of lymphocyte effector functions.

## The Fyn–ADAP Interaction: Cytotoxicity Versus Cytokine Production

**Figure 4:**
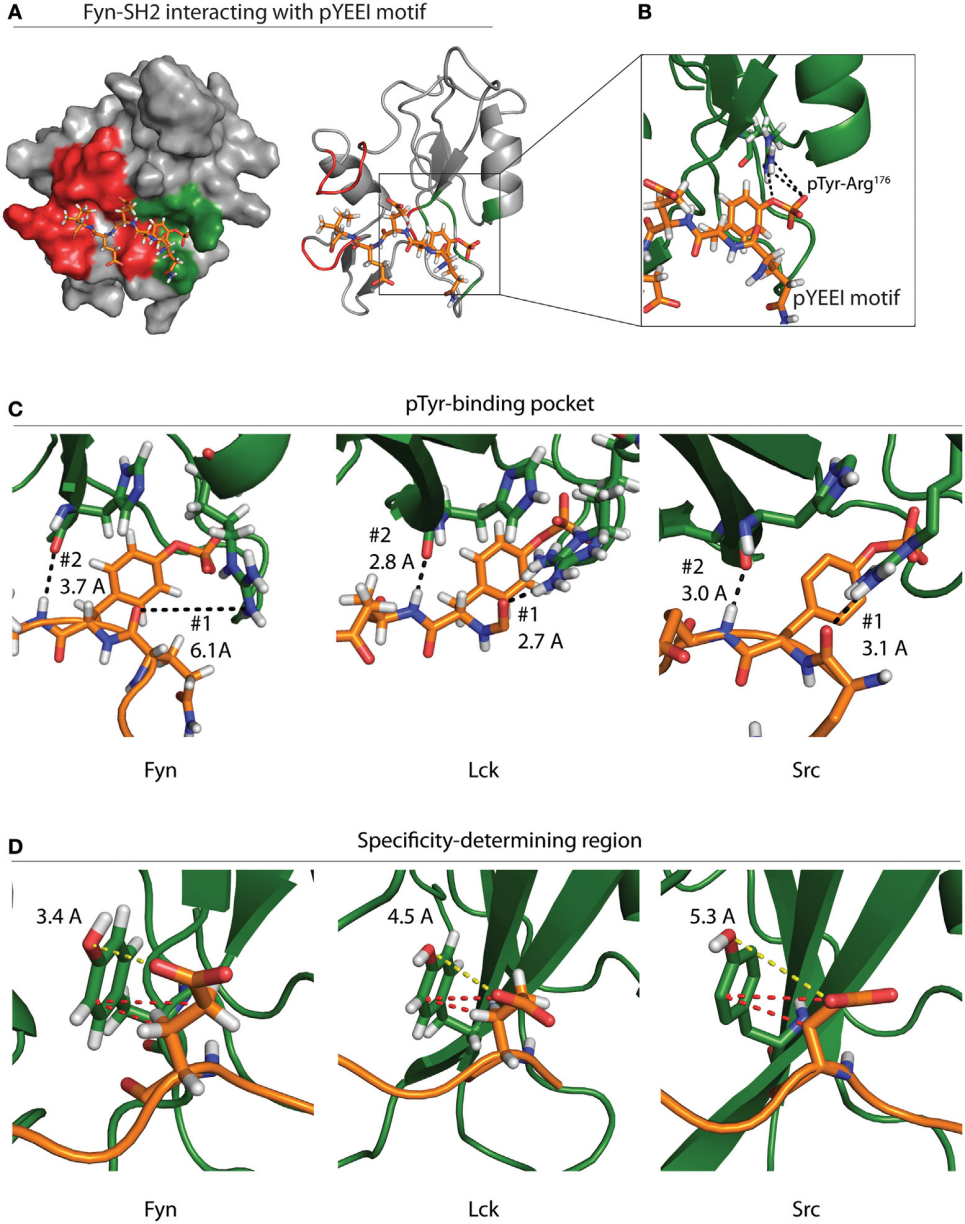
**Specificity of the Fyn–ADAP interaction**. **(A)** Surface and ribbon models show the Fyn SH2 domain bound to a pYEEI peptide motif. Green shading on the surface model represents the phosphotyrosine-binding pocket, while red shading represents the specificity-determining region. The ribbon model shows that the pYEEI peptide lies orthogonal to the β-sheet of the SH2 domain. This positions the phosphorylated tyrosine in a hydrophilic pocket in between the β-sheet and the αA helix. The linear conformation of the peptide allows residues C-terminal to the phosphotyrosine to interact with other regions of the SH2 domain, which increases specificity and affinity of the Fyn–substrate interaction. Both the surface and ribbon model are derived from PDB deposition 1AOT. **(B)** An exploded view of the primary binding pocket highlights the interaction between the phosphotyrosine and the conserved ArgβB5 of Fyn. Salt-bridge formation at this site contributes roughly 50% of the binding energy required for the SH2 peptide interaction. **(C)** Comparison of different SFKs bound to pYEEI motifs show varied interactions at the phosphotyrosine-binding pocket. Ribbon models of Fyn (left), Lck (center), and Src (right) highlight bond distances between ArgαA2 of the SH2 domain and a backbone carbonyl on the target peptide (#1). Bond distances show that ArgαA2 is capable of interacting with the backbone of the pYEEI motif in Src and Lck but not in Fyn. Ribbon models also show the distance between the backbone carbonyl of HisβD4 of the SH2 domain and backbone amide of the pYEEI motif (#2). These bond distances suggest that the structure of the Fyn SH2 domain places pYEEI motifs in a more extended conformation than that seen in Src or Lck. **(D)** Measurements obtained from PDB depositions show the distance between TyrβD5 of the SH2 domain and the glutamate residue in the +1 position from the phosphotyrosine. The shorter distance in Fyn (left) means that hydrogen bonding is possible between TyrβD5 and the +1 glutamate residue of the pYEEI motif, while bonding is based primarily on hydrophobic interactions (red dashes) in Lck (center) and Src (right). This may provide an explanation for the ability of Fyn to act as the sole kinase responsible for recruitment of ADAP, as less hydrophobic interactions would be possible between the SH2 domain and YDGI motif of ADAP. Measurements are based on PDB depositions 1AOT for Fyn, 1LKK for Lck, and 1SPS for Src.

All SFKs contain a highly conserved SH2 domain with a preference for binding pYEEI phosphopeptide motifs (56). Roughly half of the binding energy for this interaction is derived from contact between the phosphotyrosine residue and a hydrophilic binding pocket located between the N-terminal α-helix and central β-sheet of SH2 domains (Figure 4A) (27, 57). The base of this pocket is formed by an Arg residue in the β-sheet that mediates electrostatic interactions with the phosphotyrosine (Figure 4B). This pocket is conserved across SH2 domains and provides the basis for SH2–phosphotyrosine interactions (58).

SH2 domains also contain a specificity-determining region adjacent to the phosphotyrosine pocket that is required to ensure signaling integrity (Figure 4A). Variation in the specificity-determining region establishes substrate selection among SH2 domain-containing proteins. In SFKs, this region contains basic and polar residues capable of forming hydrogen bonds and electrostatic interactions with the +1 Glu residue of the pYEEI motif of substrates (56). These additional interactions provide an enthalpy-driven increase in affinity, as well as an increase in specificity for acidic residues within SFK substrate pYEEI motifs (27, 56, 57). The specificity-determining regions of SH2 domains also contain a hydrophobic pocket formed by two C-terminal loops. While the hydrophobic nature of this pocket is conserved across SH2 domains, different residue composition between protein families leads to different specificities for the +3 hydrophobic residue of target pYEEI motifs. In SFKs, a Gly residue in the C-terminal loop allows a branched chain Ile to fit stably into the hydrophobic pocket (27, 56, 57). Collectively, these interactions provide the basis for the specificity of a given SH2 domain (27, 57).

Despite the conserved specificity across SFK SH2 domains, Fyn is the only SFK that interacts with the pYDGI motif of ADAP (15). The structure of Fyn bound to the phosphotyrosine motif of ADAP remains to be elucidated. However, crystal structure of Lck and Src and NMR solution structures of Fyn bound to a model phosphotyrosine motif (pYEEI) provide a potential basis for the Fyn SH2 domain interaction with the pYDGI motif of ADAP (20). Fyn, Lck, and Src contain an Arg residue within the first α-helix (ArgαA2) located at the base of the phosphotyrosine-binding pocket. Our analyses using Protein Data Bank (PDB) depositions of Fyn, Lck, and Src bound to pYEEI-based peptides show that the different positions of this ArgαA2 may influence the conformation of the pYDGI motif in ADAP (Figures 4C,D). As noted by Campbell et al., NMR solution structures of Fyn show that the side chain of ArgαA2 (Arg^134^) has a poorly defined position, and intramolecular nuclear overhauser effect (NOE) analyses suggest that this residue does not contribute directly to phosphotyrosine binding (Figure 4C, left). Compared to Lck (Figure 4C, center) and Src (Figure 4C, right), ArgαA2 of Fyn is farther away from the backbone of the target peptide (6.1 Å in Fyn compared to 3.1 and 2.7 Å in Src and Lck, respectively) and hydrogen bonding to the corresponding main-chain carbonyl of the pYEEI motif does not occur (Figure 4C) (20). The structures of both Src and Lck show that the side chains of ArgαA2 have well-defined positions. The fixed positions of these residues combined with a shorter bond distance allows them to interact with phosphate oxygens and main-chain carbonyl groups of the pYEEI motif. Contrary to what is seen in Fyn, the structures of Src and Lck therefore identify ArgαA2 in the phosphotyrosine-binding pocket as a critical residue in positioning of the phosphotyrosine motif.

In all three structures, the main-chain carbonyl of a His in the fourth β-strand (HisβD4) interacts with backbone amide of the Glu residue in the +1 position from the phosphotyrosine (Figure 4C) (20, 27, 59). In the case of Src and Lck, the proximity of these interactions (3.0 and 2.8 Å, respectively) locks the conformation of the peptide backbone due to high energy hydrogen bonding (20, 27, 59). In the case of Fyn, the loss of pYEEI-binding to ArgαA2 corresponds to an extended distance (3.7 Å) between HisβD4 and the backbone amide of the target peptide which results in a lower energy hydrogen bond. Based on these structural analyses, we propose that the weaker bond with HisβD4 in Fyn allows the pYEEI motif to adopt a more extended conformation. This decreases the distance between a Tyr residue in the specificity-determining region of the SH2 domain of Fyn (TyrβD5) and the +1 glutamate residue of the phosphotyrosine motif (Figure 4D, left). Structural data show an increased distance between the side chain of the +1 Glu of the pYEEI motif and TyrβD5 of Lck and Src as evidenced by the distance between the Glu carboxyl group and Tyr hydroxyl group (3.4 Å in Fyn compared to 4.5 and 5.3 Å of Lck and Src, respectively) (Figure 4D) (20, 27, 59).

Previous characterization of all three structures revealed that TyrβD5 binds the +1 glutamate residue of target peptides; however, varied bond distances suggest that this contact is controlled by different interactions (20, 27, 59). The proximities of the carboxyl group of the +1 Glu to the hydroxyl group of TyrβD5 suggest that hydrogen bonding may participate in this interaction in Fyn but not in Src or Lck (20, 27, 59). In Src and Lck, this interaction is instead likely reliant upon hydrophobic interactions between the ring group of TyrβD5 of the SH2 domain and the side chain carbons of the +1 Glu of the pYEEI motif (20, 27, 59). These hydrophobic interactions may necessitate a longer side chain with an increased number of carbons in the +1 position of phosphotyrosine motifs in order to satisfy the hydrophobic interactions required to tightly bind Src and Lck substrates. This would prevent binding of peptides with shorter chain amino acids substituted in the +1 position, thereby preventing Src and Lck from interacting with the pYDGI motif of ADAP. The specificity of the Fyn–ADAP interaction may therefore enable lymphocytes to regulate proinflammatory cytokine production by limiting the amount of activated SFKs that can initiate downstream signaling cascades of ADAP.

## Conclusion

The Fyn–ADAP axis highlights the importance of structure in controlling divergent signaling cascades responsible for unique effector functions in lymphocytes. The structure of multiple SFKs, including Fyn, reveals the intramolecular interactions between the SH2 domain and C-terminal phosphotyrosine critical for regulating kinase activity. These structures in turn play an integral role in characterizing the conformational changes required to activate and inhibit SFKs. This provides mechanistic insight into both intrinsic and extrinsic regulation of Fyn. Once activated, the intramolecular organization of Fyn’s SH2 and SH3 domains allows the protein to recognize and phosphorylate multiple targets that control cellular function. Because Fyn is able to target both PI(3)K and ADAP, it is able to control cytotoxicity and cytokine production in NK cells. Data from our lab and others suggest that the ability of Fyn exclusively to activate ADAP is a result of structural differences between SFKs that allow Fyn to bind the pYDGI motif of ADAP. To date, no mechanistic basis for Fyn–ADAP specificity has been confirmed, and proving any underlying structural cause will likely require solving the structure of the Fyn SH2 domain complexed with a pYDGI containing peptide. Solving this structure has the potential to elucidate the mechanism behind the signaling specificity responsible for distinct effector functions in lymphocytes, and serve as a model for identifying other divergent signaling cascades.

## Conflict of Interest Statement

The authors declare that the research was conducted in the absence of any commercial or financial relationships that could be construed as a potential conflict of interest.
